# Anti-Inflammatory Activity of Monoterpene Phenol Dimers from *Satureja bachtiarica*

**DOI:** 10.1021/acsomega.6c03194

**Published:** 2026-09-07

**Authors:** Simone Di Micco, Milena Masullo, Noemi Marigliano, Ester Colarusso, Marzieh Rahmani Samani, Anna Schettino, Anella Saviano, Francesco Maione, Sonia Piacente, Giuseppe Bifulco

**Affiliations:** † European Biomedical Research Institute of Salerno (EBRIS), Via Salvatore De Renzi 50, Salerno 84125, Italy; ‡ Department of Pharmacy, 19028 University of Salerno, Via Giovanni Paolo II 132, Fisciano 84084, Salerno, Italy; § ImmunoPharmaLab, Department of Pharmacy, School of Medicine and Surgery, 9307 University of Naples Federico II, Via Domenico Montesano 49, Naples 80131, Italy; ∥ PhD Program in Drug Discovery and Development, Università degli Studi di Salerno, via Giovanni Paolo II n.132, Fisciano 84084, Salerno, Italy; ⊥ Nutraceuticals and Functional Foods Task Force, Department of Pharmacy, School of Medicine and Surgery, University of Naples Federico II, Via Domenico Montesano 49, Naples 80131, Italy

## Abstract

*Satureja bachtiarica* (Bakhtiari savory) is an aromatic herb cultivated both as a culinary spice and for its medicinal uses. Species of *Satureja* are known to exhibit anti-inflammatory properties. Monoterpene phenol dimers with biphenyl skeletons have been identified in these plants and structural investigations revealed the key role of the biphenyl scaffold in interacting with MAPEG family proteins. Thus, in the present contribution, we investigated by biochemical and cell-based assays, together with molecular docking and molecular dynamics investigations, the modulation of inflammatory mediators of three isolated monoterpene phenol dimers (**1**–**3**). Molecular modeling and *in vitro* enzymatic assays of **1**–**3** against soluble epoxide hydrolase (sEH) and 5-lipoxygenase (5-LOX) identified the structural elements accountable for binding vs the biological targets. Unlike **2** and **3**, *in vitro* experiments revealed that **1** did not show cytotoxicity. Interestingly, **1** selectively alters the profile of AA-derived mediators produced by LPS-activated macrophages, suppressing both IL-6 and PGE_2_. The obtained results revealed how compound **1** may not only suppress inflammation but also promote the active resolution of inflammatory responses and, by integrating computational analysis, provide clues to design more effective analogues.

## Graphical abstract

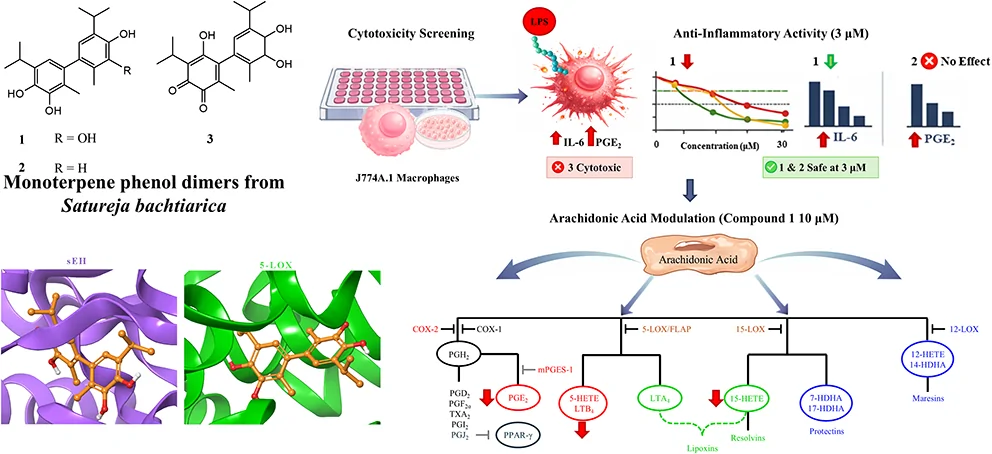

The genus *Satureja* (Lamiaceae) comprises approximately 200 species of aromatic herbs and shrubs. More than 30 of these species are found throughout the eastern Mediterranean region.^1,2^ These plants have long been employed in traditional medicine as muscle pain relievers, tonics, and carminatives to alleviate stomach and intestinal disorders. The antimicrobial, antioxidant, and anti-inflammatory activities of *Satureja* species have been widely reported in the literature.^3^ The antimicrobial properties of *Satureja* species are primarily attributed to their essential oils, which contain high levels of carvacrol, thymol and linalool. In contrast, their antioxidant capacity is mainly associated with diverse phenolic compounds, including flavonoids and other phenolic constituents. Within the nonvolatile fraction, the predominant compounds are generally rosmarinic acid together with derivatives of caffeic acid and 3,4-dihydroxyphenyl lactic acid.^4^

Species of the *Satureja* genus are valued for their diverse applications in the food, fragrance, pharmaceutical, and cosmetic sectors, largely because their essential oils are rich in the bioactive monoterpenes, thymol and carvacrol. Among them, *Satureja bachtiarica* (Bakhtiari savory) has traditionally been cultivated in Iran, where it is widely used both as a culinary spice and as a medicinal plant in folk medicine. In our previous study, we investigated the polar extract of the plant’s aerial parts and identified flavonoids, phenylpropanoids, phenolics, lignans, coumarins, triterpenes, monoterpenes, and monoterpene phenol dimers, these latter characterized by a biphenyl skeleton made up of two thymol-related moieties,^5^ differently oxidized.

Evidence from several studies indicates that extracts and essential oils of *Satureja* species exhibit anti-inflammatory activity in both *in vitro* assays, including protein denaturation, cytokine production, pro-inflammatory gene expression, and *in vivo* models such as carrageenan-induced rat paw edema.^2,4^ The effects of *Satureja hortensis* on COX-2 and iNOS expressions, demonstrating that the extract inhibited lipopolysaccharide (LPS)-induced inflammatory responses by J774A.1 macrophages, were reported.^6^ Still, only in a few cases was the chemical content of the polar extract defined, and the biological activity of isolated compounds was tested. Moreover, literature data report the anti-inflammatory activity of thymol and derivatives with the thymol-based scaffolds.^7^

Some of us widely demonstrated the importance of the bisphenol moiety for the recognition of the MAPEG protein family.^8–10^ Based on these considerations, taking into account the close similarity between the monoterpene phenol dimers and thymol, the anti-inflammatory activity of biphenyls isolated from *S. bachtiarica* was evaluated by combining *in silico* and *in vitro* studies. Specifically, we tested **1**–**3** against soluble epoxide hydrolase (sEH) and 5-lipoxygenase (5-LOX) to identify new lead compounds for the development of multitargeting modulators. The enzyme sEH hydrolyzes epoxyeicosatrienoic acids (EETs) into dihydroxyeicosatrienoic acids (DiHETrEs).^11,12^ 5-LOX converts arachidonic acid into leukotrienes (LTs).^13^ 5-LOX/sEH dual inhibition provides a twofold benefit. It suppresses the synthesis of pro-inflammatory LTs while concurrently elevating the concentration of anti-inflammatory eicosanoids (i.e., EETs).^14,15^

## Results and Discussion

### Isolation and Characterization of Monoterpene Phenol Dimers (**1–3**)

The extraction of the aerial parts of *S. bachtiarica* and the further purification process by HPLC-UV allowed us to isolate 3,4,3′,4′-tetrahydroxy-5,5′-diisopropyl-2,2′-dimethylbiphenyl (**1**), 3,4,4′-trihydroxy-5,5′-diisopropyl-2,2′-dimethylbiphenyl (**2**), and 6,3′,4′-trihydroxy-5,5′-diisopropyl-2,2’-dimethylbiphenyl-3,4-dione (**3**).

The characterization of the isolated compounds was carried out using 1D and 2D Nuclear Magnetic Resonance (NMR) experiments, along with High-Resolution Mass Spectrometry (HRMS) analyses. The combination of these analytical techniques ensured us the accurate identification and characterization^16^ of compounds **1–3**, laying the foundation for further investigations into their biological activities and potential therapeutic applications (Figure 1 and Supporting Information).

**Figure 1:**
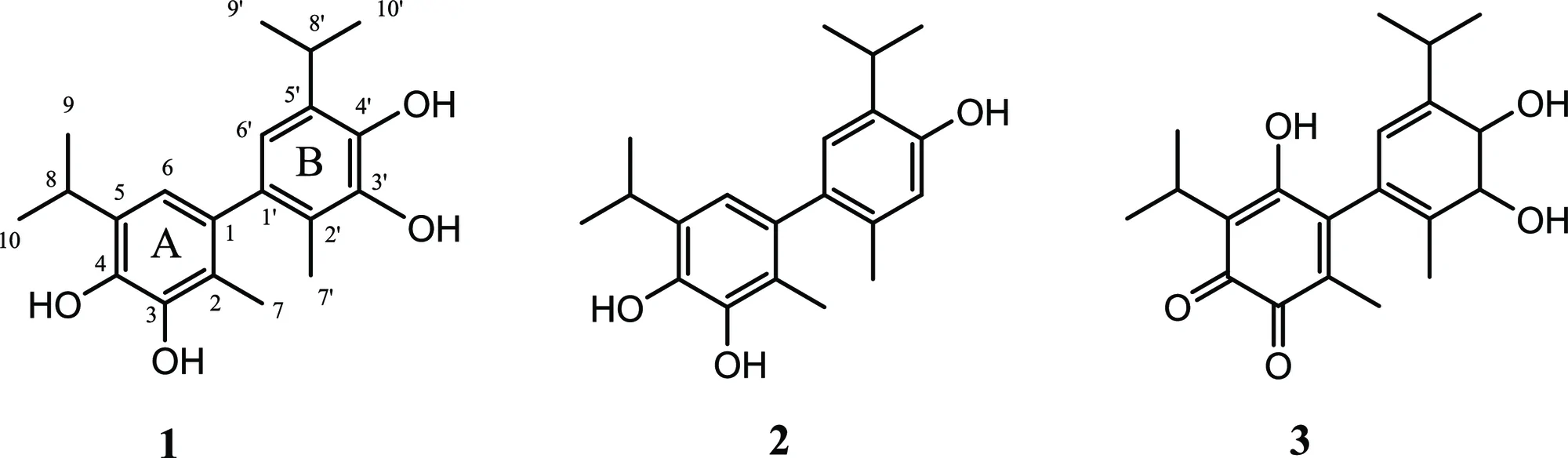
Molecular structures of monoterpene phenol dimers isolated from the aerial parts of *S. bachtiarica*.

Their formation is supposed to follow a typical phenolic reaction pathway, beginning with oxygen-catalyzed radical generation via the loss of two hydrogen atoms (one from each monoterpene molecule), enabling radical dimerization and yielding biphenyl compounds.^17^ Biphenyl compounds were also isolated from thyme leaves and were found to exhibit deodorizing, antioxidant, and inhibitory effects on human platelet aggregation.^17,18^

### Molecular Modeling and *In Vitro* Enzymatic Assays

Compounds **1–3** (Figure 1) were *in silico* screened^18^ against sEH and 5-LOX to assess their putative binding for identification of new multitargeting chemical hits, as modulators of the biosynthesis of pro-inflammatory eicosanoids. As previously applied for studies of MAPEG inhibitors,^8^ we compared the docking scores (see Experimental Section for details) of **1–3** with values achieved for a collection of 50 decoys calculated for each tested compound, similarly to our developed Inverse Virtual Screening (IVS) protocol,^19,20^ followed by visual inspection to detect plausible poses. A clear ranking was obtained from docking outcomes *vs* sEH (Table 1). With respect to their decoys, **1** and **2** distinguished themselves in terms of binding affinity, whereas **3** gave a similar docking score to the averaged values of its decoys. Indeed, compound **3** does not optimally adapt in the enzymatic pocket, giving a lower number of interactions and many steric clashes, suggesting a scarce/absent interaction with this protein.^21–23^ On the contrary, **1** and **2** showed a better placement, well-fitting the binding site of sEH. The docked pose of **1** gives a π–π T-shaped stacking by its ring A with the side chain of W525 and a parallel-displaced one with H524 (Figure 2A). The OH group at C-3 is hydrogen-bonded to the side chain of D496 and backbone NH of F497. The methyl group at C-2 establishes van der Waals contacts with V498 and H524, while the C-5 isopropyl establishes contacts with R410, L417, and W525. Ring B interacts through π-stacking with the side chain of W383 and, along with its methyl and isopropyl substituents, is engaged with van der Waals interactions with F267, Y383, F387, L408, M419, L428, Y466, H524, and W525. The OH group at C-3′ is H-bonded to the D335 side chain. The hydroxyl at C4′ donates a hydrogen bond to the Y466 side chain while accepting an aromatic H-bond from F387. These key interactions found by molecular docking investigation were preserved over time (>30%; Figure 2C), as revealed by molecular dynamics (MD) simulations (100 ns, 310 K),^24–26^ confirming their important impact on complex stability. Ring A of **2** overlaps with the same moiety of **1** but flips the accommodation of its substituents. Thus, the OH group at C-3 gives an H-bond to the main chain CO of L408 while establishing identical π–π interactions observed for **1**. The isopropyl at C-5 gives van der Waals interactions alongside M419, D496, F497, V498, and H524. Similarly, the methyl group at C-2 interacts with the side chain of L408, M419, and W525. With respect to **1**, ring B is rotated by 90° and gives π-stacking with H524. The hydroxyl group at C-4′ donates a hydrogen bond to D335 and accepts one from Y466. The isopropyl of ring B presents van der Waals contacts with D335, Y383, Q384, V498, Y466, and L499. The same interactions are provided by the methyl substituent at C-2′ with P268, H524, and W525. Noteworthy, most of the observable contacts for **1** and **2** are given by the cocrystallized ligands with sEH.^27,28^

**Table 1:** V Values Obtained from Molecular Docking (See Methods). Percentage of sEH and 5-LOX inhibition and IC_50_. Data are reported as a percentage respect to the control (100%, *n* = 3).

	**sEH**				5-LOX			
**compound**	**V**	**% of inhibition at 10 μM**	**% of inhibition at 1 μM**	**IC**_**50**_ **± SD** **(μM)**	**V**	**% of inhibition at 10 μM**	**% of inhibition at 1 μM**	**IC**_**50**_ **± SD** **(μM)**
**1**	1.32	85 ± 7	49 ± 6	0.66 ± 0.06	1.53	65 ± 3	50 ± 1	1.1 ± 0.2
**2**	1.24	52 ± 2	27 ± 3	8.5 ± 0.6	1.30	62 ± 1	43.3 ± 1.2	2.9 ± 0.2
**3**	1.11	27 ± 8	25 ± 3	\	0.72	13 ± 2	1.5 ± 2.3	\

**Figure 2:**
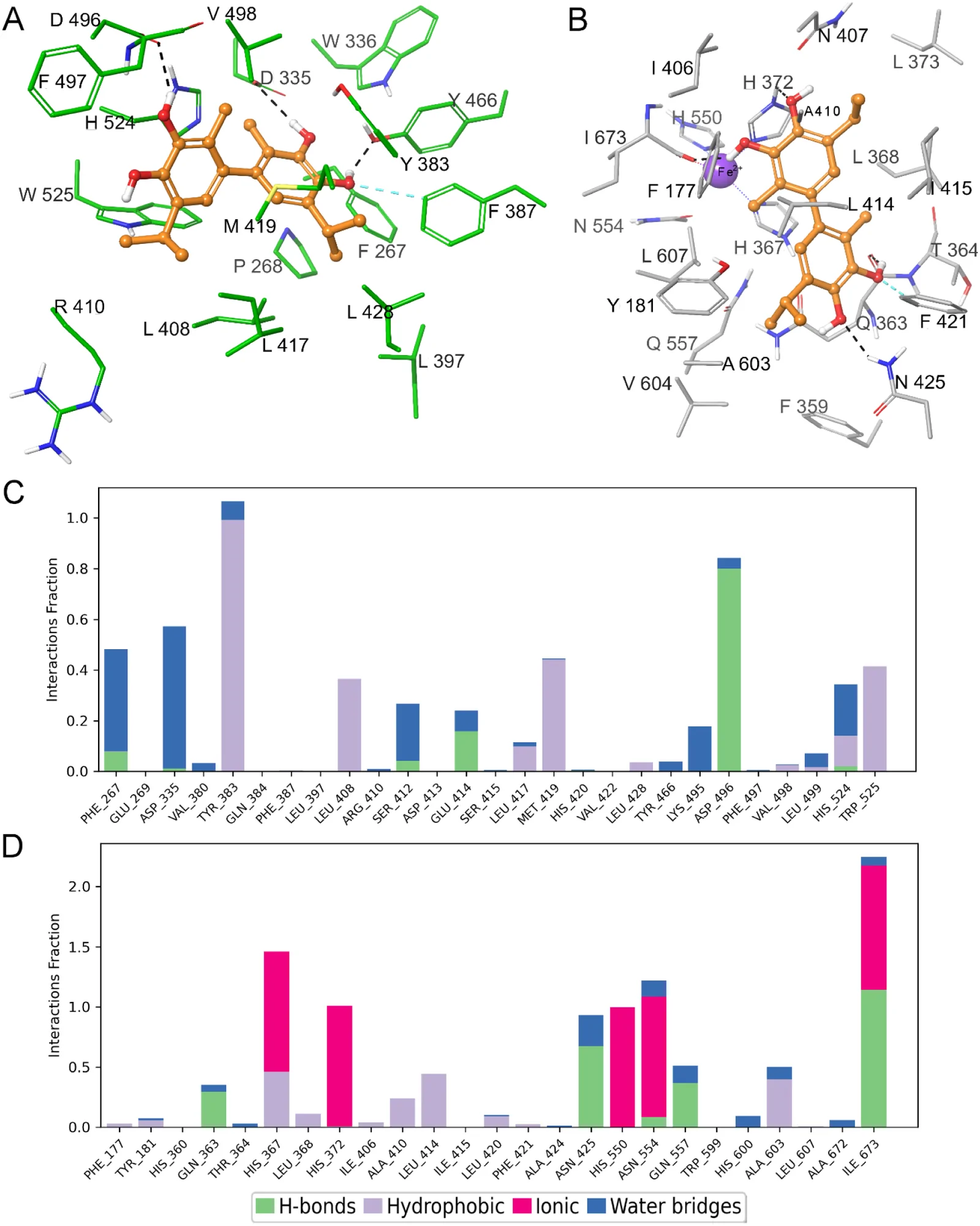
3D mode of the interactions established by **1** with sEH (A) and 5-LOX (B). sEH and 5-LOX are, respectively, depicted by green (A) and gray (B) tubes with the atom color code as follows: C, as for the tube; polar H, white; N, dark blue; O, red; S, yellow. The ligand is sketched by orange sticks and balls (color coded as the proteins but for C like sticks). (B) Fe^2+^ is sketched by violet cpk. The dashed black and cyan lines specify, respectively, ligand-protein hydrogen and aromatic H-bonds. Contact histograms from MD simulations of **1** with sEH (C) and 5-LOX (D).

About the screening toward 5-LOX, **1** appeared top-ranked, followed by **2** that also performed better than their relative decoys. On the contrary, **3** displayed a lower docking score than the decoy average. Indeed, **3** showed a poor fit with the enzymatic pocket. Ring A of **1** gives a π-stacking with H372 and van der Waals interactions with F177, L368, A410, I415, and L414 (Figure 2B). Similar contacts are also provided by the isopropyl substituent of C-5 with side chains of L368, H372, L373, N407, and I415. Analogously, the methyl group at C-2 binds with F177, H367, L414, and L607. The OH at C-3 donates an H-bond to I673 main-chain CO, while the hydroxyl at C-4 is hydrogen-bonded to the branched chains of H372 and N407. OH at C-3′ is engaged with a hydrogen bond and an aromatic bond with the main-chain CO of Q363 and F421, respectively. The same substituent of C-4 is bound to the N425 branched chain. Ring B and its alkyl substituents make van der Waals bonds with Y181, N363, L368, L414, I415, F421, and A603. As found for **1**-sEH, MD trajectory analysis showed the stability of the complex formed with 5-LOX over time (Figure 2D). Compound **2** establishes two hydrogen bonds by its catechol moiety with the backbone CO of I673. Ring A and its alkyl groups interact through van der Waals contacts with F177, H367, H372, N407, A410, L414, F421, and L607. Similarly, ring B and hydrophobic substituents are engaged in van der Waals interactions with W147, F151, L368, L373, N407, A410, R411, I415, and F421. The C-4′ hydroxyl receives a hydrogen bond from the W147 side chain. Moreover, we also investigated the putative binding of **1**–**3** into the allosteric site of 5-LOX.^29^ The theoretical outcomes suggested that **1**–**3** are not suitable allosteric binders, especially for the unproper positioning of methyl and isopropyl groups, respectively, against R101 and H130, giving steric clashes and a bent of the biphenyl moiety (Figure S1). Polar interactions with R101 and H130 are fundamental for binding in this enzyme region, as shown for the well-known allosteric inhibitor AKBA.^30^ Collectively, the theoretical screening suggested that compounds **1** and **2** are potential binders of sEH and 5-LOX, unlike **3**.

Once isolated and purified, **1**–**3** were tested for their inhibition *vs* sEH and 5-LOX to experimentally validate the theoretical outcomes. First, each compound was tested on sEH enzyme at two different concentrations, 10 μM and 1 μM (Table 1), using a fluorometric cell-free assay (see Methods for details). Notably, in line with theoretical analysis, **1** demonstrated greater efficacy, with a stronger inhibitory effect at both concentrations used. Specifically, **1** overcame 80% of inhibition at 10 μM and reached 50% at 1 μM (Table 1). Analog **2** also significantly inhibited the enzymatic activity of sEH (52 ± 2%, Table 1) at 10 μM, but a lower efficacy was shown at 1 μM. As predicted by the *in silico* investigation, **3** did not display a significant inhibitory effect on sEH activity at the two tested concentrations, indicating poor or absent interaction with the enzymatic target. Based on these promising preliminary results, the potency of both **1** and **2** was further investigated by determining their IC_50_ values (Table 1 and Figure S5) to better characterize their inhibition profiles. As expected, the best IC_50_ value was obtained for **1** (Table 1 and Figure S5).

We extended our analysis to assess the 5-LOX inhibitory activity of derivatives **1–3**. To verify their ability to interfere with leukotriene biosynthesis, we performed a UV-based assay, which showed that compounds **1** and **2** exhibited comparable activity (Table 1) at both tested concentrations. Notably, **1** emerged as the most potent compound, showing a roughly threefold higher inhibitory effect compared to **2**, as shown by their respective IC_50_ values: 1.1 ± 0.2 μM and 2.9 ± 0.2 μM (Table 1 and Figure S6). In contrast, **3** did not exhibit any appreciable inhibitory activity against 5-LOX. Finally, since our goal is to identify selective sEH/5-LOX inhibitors without affecting cyclooxygenases (COXs) and inducing the consequent side effects, the natural products (**1**–**3**) were assessed on COX-1 and COX-2. The cell-free enzymatic assays (Table S1) demonstrated poor inhibition of both COX isoforms by **1**–**3** under the conditions employed. Notably, the activity of the three compounds is slightly better at 1 μM than at 10 μM because a slight precipitation effect is observed at 10 μM, which is not present at 1 μM under experimental conditions. We rationalized these results by means of a molecular modeling investigation.^9^ We did not observe plausible docked poses in the binding sites of both COXs.

### *In Vitro* Characterization of Monoterpene Phenol Dimers in Murine Macrophages

To investigate the potential anti-inflammatory properties of the three monoterpene phenol dimers (**1–3**), we first assessed their cytotoxic profiles in J774A.1 murine macrophages. Cells were plated in 96-well plates and incubated for 24 h with increasing concentrations (1–30 μM) of each compound. Viability was assessed using the MTT assay, which relies on the ability of metabolically active cells to reduce MTT into insoluble formazan crystals through mitochondrial dehydrogenase activity (Figure 3A).^31,32^ This colorimetric method is widely employed for assessing cell viability and cytotoxicity in adherent cell lines.^33^ A threshold of 75% cell viability (denoted by the dotted line) was established to define noncytotoxic concentrations for subsequent mechanistic studies (Figure 3, B–D). Based on this criterion, compound **3** exhibited significant cytotoxicity even at the lowest concentration tested and was therefore excluded from further analyses. In contrast, compounds **1** and **2** displayed a concentration-dependent reduction in viability, with 3 μM identified as the highest noncytotoxic concentration common to both compounds.

**Figure 3:**
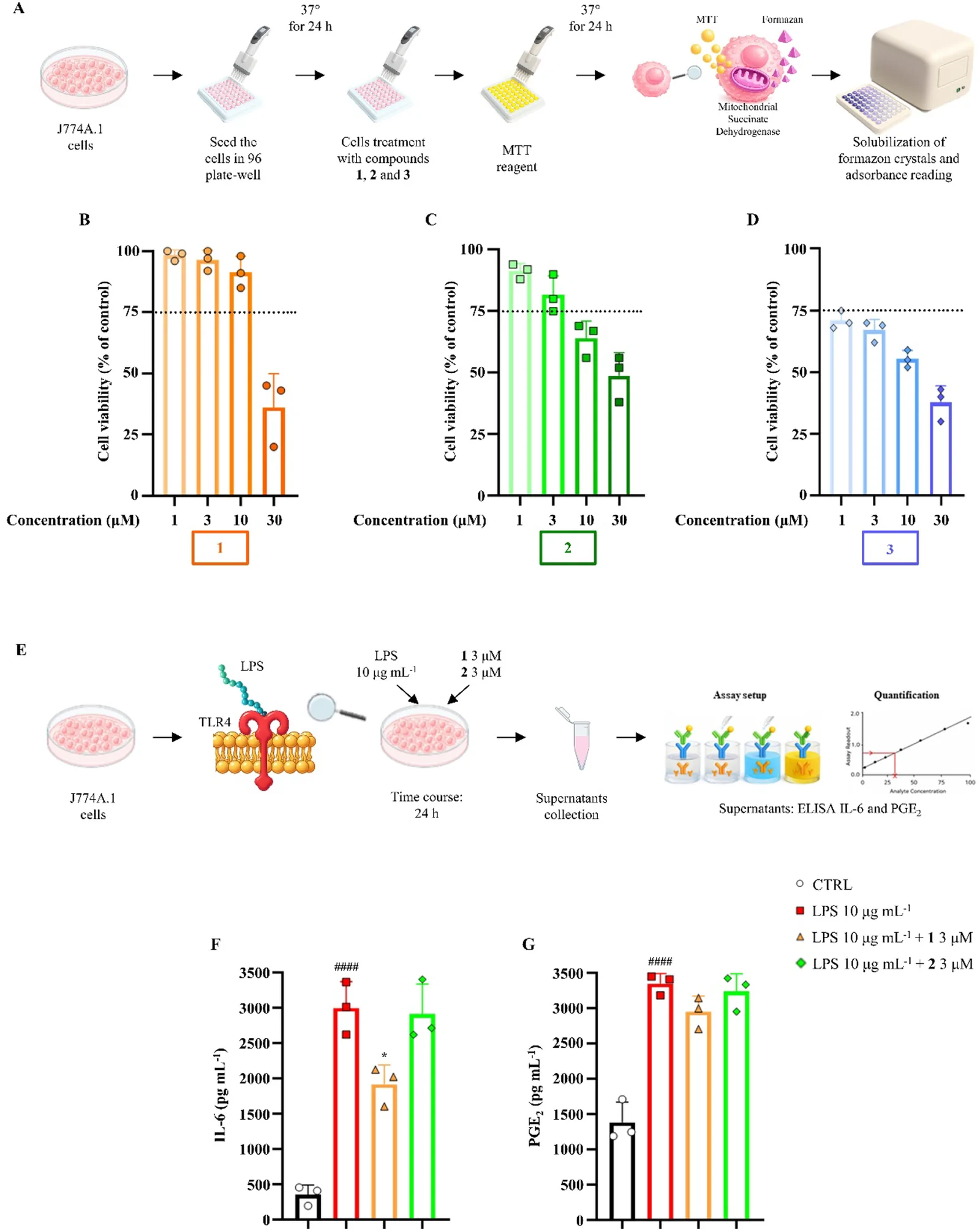
(A–D) *In vitro* mechanistic characterization of **1** and **2** in murine macrophages. Cell viability was evaluated by MTT assay in J774A.1 cells upon 24 h exposure to **1**–**3** (1–30 μM). Vehicle-treated cells (0.1% DMSO, the highest concentration) served as controls. The dotted line denotes the 75% cell viability threshold adopted to define noncytotoxic concentrations. Data are expressed as the percentage of viable cells relative to the control and presented as means ± SD of 3 independent experiments. (E–G) Effects of **1** and **2** on LPS-induced inflammatory mediator release. J774A.1 cells were cotreated with **1** and **2** at 3 μM (the highest noncytotoxic concentration identified) and LPS (10 μg mL^–1^) for 24 h. IL-6 and PGE_2_ concentrations in culture supernatants were measured by ELISA. Data are reported as pg mL^–1^ and presented as means ± SD of 3 independent experiments. Statistical analysis was conducted by one-way ANOVA followed by Bonferroni’s *post hoc* test for multiple comparisons. ^####^ *P* ≤ 0.0001 *vs* CTRL group; *P ≤ 0.05 *vs* LPS group.

We subsequently assessed the ability of compounds **1** and **2** to modulate LPS-driven inflammatory responses in J774A.1 macrophages. Cells were co-treated with compounds **1** or **2** (3 μM) and LPS (10 μg mL^–1^) for 24 h. Consistent with its pro-inflammatory activity, LPS markedly increased IL-6 and PGE_2_ production relative to unstimulated controls ^(####^ *P* ≤ 0.0001). Notably, treatment with compound **1** significantly attenuated IL-6 release (*P ≤ 0.05 *vs* LPS group) and induced a reduction in PGE_2_ levels, although this effect was not statistically significant. Conversely, **2** failed to modulate the release of either mediator at this concentration, indicating a superior anti-inflammatory activity of **1** (Figure 3, E–G). These data suggest that compound **1**, but not compound **2**, possesses anti-inflammatory properties by suppressing key cytokine and lipid mediator production in activated macrophages.

### Compound 1 Modulates the Arachidonic Acid Cascade and Lipid Mediator Production in LPS-Stimulated Macrophages

To gain deeper mechanistic insights into the anti-inflammatory activity of compound **1**, we investigated its effects on the arachidonic acid (AA) metabolic pathway, a central hub in the inflammatory response that generates both pro-inflammatory and pro-resolving lipid mediators (Figure 4A).^34^ The concentrations of cytokines and eicosanoids in culture supernatants were quantified by a highly sensitive enzyme-linked immunosorbent assay (ELISA), a well-established method for the sensitive and specific detection of inflammatory mediators. For these experiments, a concentration of 10 μM for compound **1** was employed, which was confirmed to be non-cytotoxic in preliminary experiments.

**Figure 4:**
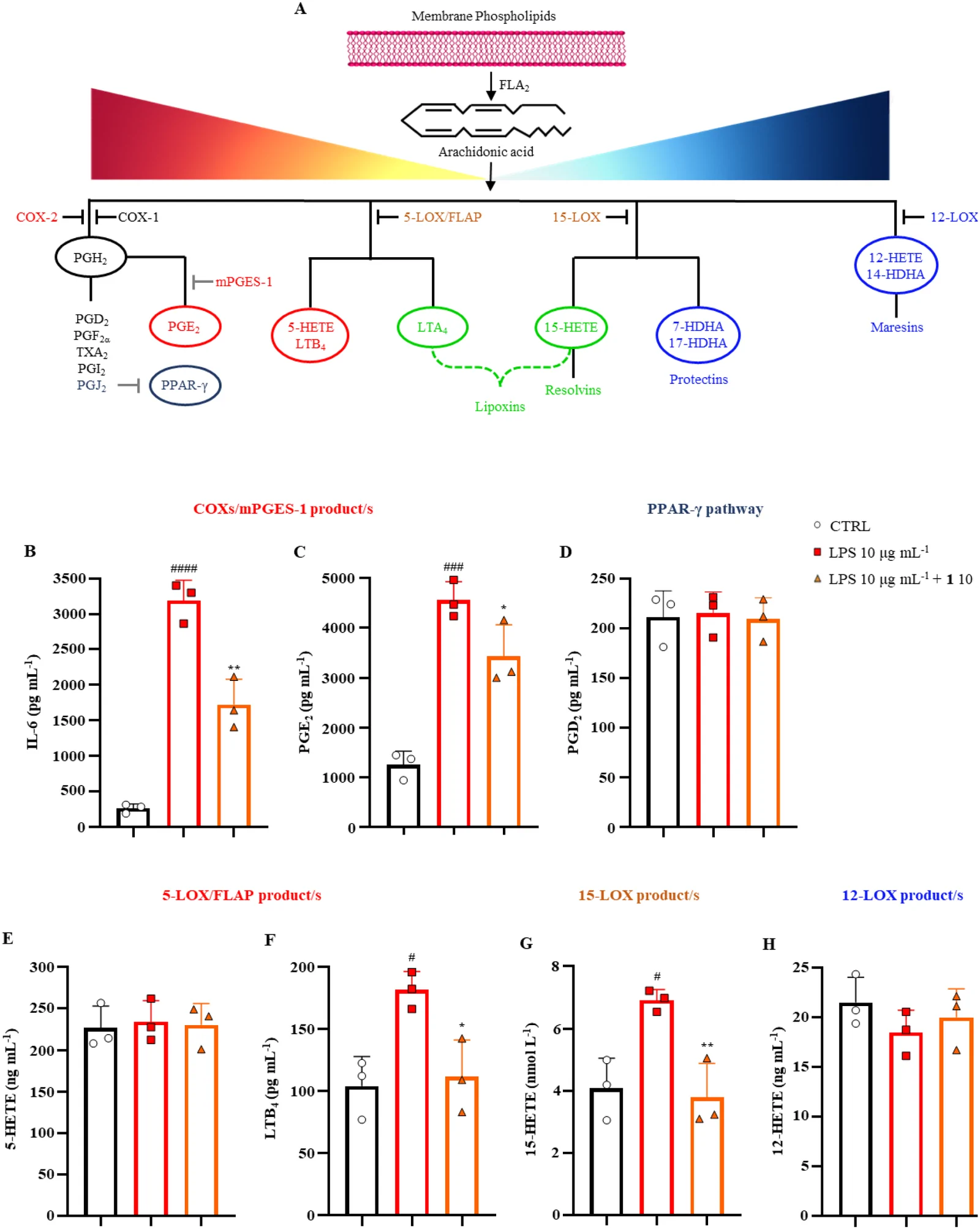
Compound **1** modulates the arachidonic acid cascade and lipid mediator production in LPS-stimulated macrophages. (A) Schematic representation of the arachidonic acid (AA) metabolic pathway. (B–H) Effects of compound **1** on LPS-induced COX- and LOX-derived lipid mediators. J774A.1 cells were cotreated with compound **1** (10 μM, the highest noncytotoxic concentration identified) and LPS (10 μg mL^–1^) for 24 h. IL-6, PGE_2_, PGD_2_, 5-HETE, LTB_4_, 15-HETE, and 12-HETE concentrations in culture supernatants were measured by ELISA. Data are reported as pg mL^–1^ (IL-6, PGE_2_, PGD_2_, and LTB_4_), ng mL^–1^ (5-HETE and 12-HETE), or nmol L^–1^ (15-HETE) and presented as means ± SD of 3 independent experiments. Statistical analysis was conducted by one-way ANOVA followed by Bonferroni’s *post hoc* test for multiple comparisons. ^#^ *P* ≤ 0.05, ^###^ *P* ≤ 0.001, ^####^ *P* ≤ 0.0001 *vs* CTRL group; *P ≤ 0.05, **P ≤ 0.01 *vs* LPS group.

Strikingly, treatment with compound **1** (10 μM) profoundly and selectively altered the profile of AA-derived mediators produced by LPS-activated macrophages (Figure 4, B–H). Compound **1** significantly decreased IL-6 production (**P ≤ 0.01 *vs* LPS). Regarding cyclooxygenase (COX) pathway metabolites, compound **1** significantly reduced PGE_2_ levels (*P ≤ 0.05 *vs* LPS), consistent with our initial observations at the lower concentration. However, **1** did not significantly modulate the production of PGD_2_, suggesting a differential effect on the terminal synthases downstream of COX. Notably, compound **1** exerted a marked inhibitory effect on the lipoxygenase (LOX) pathways. It significantly reduced the levels of LTB_4_, a potent chemoattractant produced by the 5-LOX pathway (*P ≤ 0.001 *vs* LPS). In contrast, **1** did not significantly modulate the production of 5-HETE or 12-HETE. Interestingly, a significant reduction was observed for 15-HETE, a metabolite generated by the 15-LOX pathway (**P ≤ 0.01 *vs* LPS), indicating a robust inhibitory effect (Figure 4G).

The initial screening of monoterpene phenol dimers (**1**–**3**) in J774A.1 murine macrophages revealed marked differences in both their cytotoxic profiles and their ability to modulate LPS-induced inflammatory responses, providing important insights into structure–activity relationships and guiding the selection of the most promising compound for further mechanistic investigation.

The MTT assay, a gold-standard method for assessing cell viability based on mitochondrial metabolic activity,^33^ demonstrated that compound **3** exhibited significant cytotoxicity even at the lowest concentration tested (1 μM), rendering it unsuitable for anti-inflammatory studies. This pronounced cytotoxic effect may be attributed to the specific substitution pattern of the benzoquinone core in compound **3**.

In contrast, compounds **1** and **2** displayed a more favorable safety profile, with 3 μM identified as the highest noncytotoxic concentration common to both compounds. This concentration was therefore selected for subsequent immunomodulatory studies to ensure that any observed anti-inflammatory effects were not confounded by underlying cytotoxicity, a critical consideration in the pharmacological evaluation of potential anti-inflammatory agents.^31^ When evaluated at the equitoxic concentration of 3 μM, compounds **1** and **2** exhibited strikingly different immunomodulatory activities. While both compounds were non-cytotoxic at this concentration, only compound **1** effectively blunted the LPS-driven release of IL-6 and PGE_2_. The failure of compound **2** to modulate either mediator at the same concentration indicates that the anti-inflammatory activity is not a class effect common to all monoterpene phenol dimers but rather depends on specific structural features present in **1** but absent in **2**.

The differential activity between compounds **1** and **2** is particularly intriguing, given their structural similarity. This observation suggests that subtle differences in substitution patterns can profoundly influence the interaction with molecular targets involved in inflammatory signaling. Previous studies on structurally related polyphenolic compounds similarly demonstrated that hydroxyl and methoxy group positioning profoundly affects both target affinity and downstream biological activity.^35^ It is plausible that compound **1** possesses a more favorable configuration for engaging key regulatory proteins, such as kinases involved in NF-κB activation or enzymes of the arachidonic acid cascade, compared to its compound **2** counterpart.

The ability of compound **1** to suppress both IL-6 and PGE_2_ is of particular therapeutic relevance. IL-6 is a pleiotropic cytokine with central roles. It is implicated in the initiation and maintenance of chronic inflammatory conditions, including rheumatoid arthritis, inflammatory bowel disease, and atherosclerosis.^36^ The clinical success of tocilizumab, a monoclonal antibody targeting the IL-6 receptor, underscores the importance of this cytokine as a druggable target in inflammatory disorders.^37^ Similarly, PGE_2_ is a major pro-inflammatory prostaglandin responsible for fever, pain, and vasodilation, and its biosynthesis is primarily driven by the inducible COX-2 and mPGES-1 enzymes in activated macrophages.^38^

The differential modulation of the AA cascade by compound **1** provides important insights into its mechanism of action. The reduction of PGE_2_, without concomitant inhibition of PGD_2_, is particularly noteworthy. PGE_2_ is a major pro-inflammatory prostaglandin synthesized from PGH_2_ by microsomal prostaglandin E synthase-1 (mPGES-1), while PGD_2_ is produced by hematopoietic PGD synthase (HPGDS) and can give rise to anti-inflammatory cyclopentenone prostaglandins of the J series.^39^ Recent work by *ImmunoPharmaLab* and other colleagues has established the existence of a functional “mPGES-1/PPARγ axis,” whereby the coordinated regulation of mPGES-1 and peroxisome proliferator-activated receptor γ (PPARγ) controls the shift from pro-inflammatory PGE_2_ to anti-inflammatory PGD_2_/PGJ_2_ during the resolution of inflammation.^40,41^ Further studies from this research group have demonstrated that this axis can be pharmacologically modulated by natural products, leading to superior anti-inflammatory effects.^42,43^ The selective inhibition of PGE_2_ by compound **1**, in the absence of PGD_2_ modulation, suggests that compound **1** may target mPGES-1 rather than upstream COX enzymes, thereby preserving the potential for PGD_2_-mediated pro-resolving signaling. This is further supported by the observation that compound **1** reduced 15-HETE, a product of 15-LOX that has been implicated both in pro-inflammatory signaling and as a precursor to specialized pro-resolving mediators (SPMs) such as lipoxins. Indeed, 15-LOX is strongly upregulated in alternatively activated M2 macrophages and plays a critical role in the biosynthesis of SPMs.^44^ The reduction of LTB_4_, a potent neutrophil chemoattractant, further underscores the anti-inflammatory potential of compound **1**, as LTB_4_ is a key driver of acute inflammatory responses. Collectively, these results demonstrate that compound **1** exerts a multifaceted anti-inflammatory effect by selectively modulating specific branches of the AA cascade, potentially through interference with the mPGES-1/PPARγ axis and selective inhibition of 5-LOX and 15-LOX pathways, while sparing the PGD_2_ synthetic pathway. This profile suggests that compound **1** may not only suppress inflammation but also promote the active resolution of inflammatory responses.

Taken together, these findings support further investigation of these compounds as potential leads for the development of novel anti-inflammatory drugs with a safer pharmacological profile.

## Conclusions

Overall, the substances examined in this work exhibited a dual inhibitory profile, showing noteworthy efficacy against both 5-LOX and sEH. Interestingly, these compounds showed no discernible inhibition of COX-1 or COX-2, indicating a specific mode of action. This selectivity is especially beneficial because it may reduce the likelihood of cardiovascular and gastrointestinal adverse effects that are frequently associated with COX inhibition. Structure–activity analysis suggested that both apolar and polar moieties are key structural features for the binding against sEH and 5-LOX. Indeed, both rings and their alkyl substituents are engaged in π–π and van der Waals interactions, while hydroxyl and carbonyl groups establish an H-bond network. It is noteworthy that the composition of the polar groups could address the affinity and selectivity against the biological targets. The catechol of cycle B raises the affinity *vs* sEH, especially for the orientation of the ring and the consequent established favorable contacts. Interestingly, inhibitory activity against sEH is often obtained by compounds endowed with amide and urea groups interacting with the catalytic triad of the enzyme. Here, we showed that our compounds are nitrogen-free but can block sEH. These structural features are in agreement with previously reported studies on polyphenols,^45–47^ as inhibitors of this biological target. In contrast, the absence of the hydroxyl at C-3′ is less impactful for the recognition of 5-LOX. The switch from a catechol to two carbonyl groups entails the presence of just acceptors of H-bonds, reducing the number of contacts. This structural aspect adds to the further OH substituent at C-6, hampering an appropriate fitting into the macromolecular pocket. Compounds **1** and **2** are predicted, based on structural analyses, to bind within the catalytic cavity of 5-LOX in a manner consistent with the cocrystallized active-site inhibitor NDGA and other polyphenols, rather than acting through an AKBA-like allosteric mechanism. Moreover, literature evidence^29^ indicates that AKBA may induce alterations in lipoxygenase product profiles, potentially via allosteric modulation, whereas NDGA inhibits the formation of both 12- and 15-HETE through direct binding to the active site. Our experimental results, showing selective suppression of 15-HETE without affecting 12-HETE levels in J774A.1 cells, are consistent with a catalytic-site binding mode for compound **1**. Nevertheless, further investigations will be necessary to elucidate its precise binding mode within the enzyme.

This study provides a comprehensive *in vitro* characterization of monoterpene phenol dimers in murine macrophages, identifying **1** as a promising anti-inflammatory lead compound. Initial cytotoxicity screening revealed that compound **3** was overtly cytotoxic, while **1** and **2** exhibited acceptable safety profiles up to 3 μM. At this concentration, compound **1**, but not **2**, significantly suppressed LPS-induced IL-6 and PGE_2_ release, demonstrating structural specificity and identifying **1** as the most active derivative.

Mechanistic investigation of the arachidonic acid (AA) cascade revealed that compound **1** (10 μM) selectively inhibits key inflammatory mediators. Notably, **1** significantly reduced IL-6, PGE_2_, LTB_4_, and 15-HETE, while sparing PGD_2_, 5-HETE, and 12-HETE. The reduction of LTB_4_, a potent neutrophil chemoattractant and key driver of acute inflammation, further underscores its anti-inflammatory potential. Collectively, these results demonstrate that compound **1** exerts a multifaceted effect by selectively modulating specific branches of the AA cascade, potentially through interference with the mPGES-1/PPARγ axis and inhibition of 5-LOX and 15-LOX pathways, while preserving PGD_2_ synthesis, a profile suggesting that **1** may not only suppress inflammation but also promote active resolution.

This study is limited by the absence of *in vivo* validation, the lack of definitive molecular target identification, and the need for confirmation in primary human cells. Future studies should focus on (i) evaluating compound **1** in relevant animal models of inflammation; (ii) identifying its precise molecular targets through chemical proteomics and binding assays; and (iii) exploring structure–activity relationships through analog synthesis to optimize potency and selectivity.

In conclusion, **1** represents a likely candidate for future advancement as an anti-inflammatory compound. Its ability to simultaneously target multiple inflammatory pathways, coupled with its favorable cytotoxicity profile and potential pro-resolving properties, positions it as a valuable tool for probing inflammatory signaling and a potential lead for novel therapeutics targeting chronic inflammatory diseases. From a translational *in vivo* perspective, the IC_50_ values of compound **1** against sEH (0.66 ± 0.06 μM) and 5-LOX (1.1 ± 0.2 μM) fall within the range of known bioactive natural products that display efficacy in rodent inflammation models after oral or intraperitoneal administration (typical doses 1–30 mg kg^–1^). Considering a molecular weight of 358.5 g mol^–1^ for **1**, the IC_50_ concentrations correspond to approximately 0.24–0.38 μg mL^–1^, which are theoretically achievable in plasma and tissues. Nevertheless, definitive proof of *in vivo* efficacy will require pharmacokinetic and pharmacodynamic studies in animal models of acute and/or chronic inflammation, which we plan as a follow-up investigation.

## Experimental Section

### Extraction and Isolation of Compounds **1–3**

The aerial parts of *Satureja bachtiarica* Bunge were collected in Chaharmahal va Bakhtiari Province, Iran. Botanical identification was performed by Dr. H. A. Shirmardi, Ph.D. (Research Center of Agriculture and Natural Resources, Shahrekord, Iran). A voucher specimen (No. 3621) was deposited in the herbarium of the same research center.

The collected plant material (50 g) was air-dried for 5 days in the shade at 30 ± 2 °C and extracted with ethanol/water (70:30, v/v). Evaporation of the solvent afforded 5.17 g of crude extract, which was subsequently partitioned between *n*-BuOH and water (10 mL) to eliminate free sugars, following the procedure described by Samani *et al.* (2023). This step yielded 3.17 g of the *n*-BuOH fraction. A 3 g portion of the *n*-BuOH extract was chromatographed on a Sephadex LH-20 column (100 × 5 cm; GE Healthcare, Sigma-Aldrich, Milan, Italy) using methanol as the eluent. A total of 82 fractions (10 mL each) were collected and examined by TLC. Fractions exhibiting similar chromatographic profiles were pooled, resulting in 14 major fractions. Selected fractions were further purified by reversed-phase HPLC coupled with UV detection. Separations were carried out using water containing 0.1% formic acid (solvent A) and acetonitrile containing 0.1% formic acid (solvent B) at a flow rate of 2.0 mL min^–1^. Chromatograms were monitored at 254 nm, and all analyses were performed at room temperature. Fraction 6 (205 mg) was initially subjected to HPLC-UV using a gradient starting at 5% solvent B and reaching 100% B over 30 min, yielding compound **1** (3.5 mg; RT = 26.2 min) and compound **2** (2.8 mg; RT = 28.5 min). A second purification of the same fraction was performed with a linear gradient from 5% to 20% B over 5 min, followed by 20% to 60% B over 20 min, 60% to 85% B over 5 min, and finally 85% to 100% B over 10 min. This procedure afforded compound **3** (2.5 mg; RT = 34.2 min). The collected HPLC peaks were concentrated under reduced pressure and dried prior to NMR characterization. The isolated compounds exhibited a purity greater than 99%, as determined by HPLC analysis.

### General Methods

NMR experiments were performed in methanol-d_4_ (99.95%, Sigma-Aldrich, Milan, Italy) on a Bruker DRX600 spectrometer (Bruker BioSpin GmBH, Rheinstetten, Germany) equipped with a Bruker 5 mm PATXI probe at 300 K (Figures S2–S4). TopSpin 3.2 software was used to process the data. Semipreparative HPLC-UV was performed with a Phenomenex C_18_ Synergy-Hydro-RP (25 cm × 10 mm, 10 μm) on an Agilent 1260 Infinity system (Agilent Technologies, Palo Alto, CA, USA), equipped with a binary pump (G-1312C), a UV detector (G-1314 B), and a Rheodyne injector with a loop of 20 μL.

### Molecular Docking

The three-dimensional structures of **1**–**3** were sketched through the Build Panel of Maestro (version 14.3)^48^ and optimized using the MacroModel module^49–51^ in the following manner: Polak–Ribiere conjugate gradient minimization method (maximum derivative <0.001 kcal/mol), OPLS4 force field,^52^ GB/SA (generalized Born/surface area)^53^ to model H_2_O as solvent. Then, **1**–**3** were managed by LigPrep, producing protonation states at pH = 7.0 ± 1.0.^54,55^ The X-ray crystallographic structures of sEH (PDB ID: 3i28),^10^ catalytic site 5-LOX (PDB ID: 3O8Y),^56^ 5-LOX allosteric site (PDB ID: 6NCF),^29^ COX-1 (PDB ID: 1Q4G),^57^ and COX-2 (PDB ID: 5IKR)^58^ were employed as biological target models and managed through the Protein Preparation Wizard:^59,60^ checks for missing side chains/loops and residue alternate positions; assignment of bond orders; addition of hydrogens ; assignment of side chain charges based on their p*K*_a_ at pH 7.0. The optimize option was adopted to refine the H-bond network. Co-crystallized ligands and molecules of water were deleted. Molecular docking was accomplished through Glide.^49,61–64^ The conformational search parameters used for molecular docking were previously reported for sEH and 5-LOX. In particular, for the 5-LOX investigation, we applied the Induced Fit Docking (IFD) approach, accounting for protein cavity shaping upon ligand binding.^65–69^ To validate our docking methodology for the COXs and the 5-LOX allosteric site, we docked^70–73^ the cocrystallized inhibitors, obtaining an RMSD of 0.26 Å for (2-(1,1′-biphenyl-4-yl)­propanoic acid) BFL with COX-1, 0.27 Å for ID8 (2-[(2,3-dimethylphenyl)­amino]­benzoic acid) with COX-2, and an RMSD of 1.17 Å for AKBA with the 5-LOX allosteric site. Inner (10 Å) and outer (13 Å) receptor grid boxes were applied between chains A and C of COX-1 with x, y, and z coordinates: −21.18, −6.39, −30.48. For COX-2, the grids were set at 10 (inner) and 16 Å (outer) and placed at the chain A–C interface: −20.02 (x), 7.71 (y), 30.74 (z). Concerning the 5-LOX allosteric site, the grid dimensions were 10 Å (inner) and 18 Å (outer), centered at: 10.32 (x), −22.52 (y), −19.09 (z). First, one pose/ligand was generated using the default parameters of SP (Standard Precision). The so-generated conformations were employed as input for an XP (Extra Precision) Glide mode round of predictions. Then, the outcomes were used for a second run of calculations and similarly to obtain a third round. By applying the enhanced sampling mode, 10^4^ poses per ligand were maintained for the first docking step, 1,600 poses per ligand were picked for energy minimization, and a maximum of 1,000 output structures was generated for each ligand. The partial charge cutoff and the scaling factor for van der Waals radii were set at 0.15 and 0.8, respectively. The docked poses were optimized, maintaining a maximum of 100 conformations, with a cutoff of 0.5 kcal/mol for rejecting minimized poses. In the energy score, the following contributions were also accounted: reward of intramolecular H-bonds; aromatic H-bonds; Epik state penalty. As formerly described,^74^ the calculated docking scores were normalized by means of the equation: V = V_**0**_/V_**R**_, where V_**0**_ is the docking score (kcal/mol) of each small molecule (**1**–**3**) and V_**R**_ is the average value (kcal/mol) among the decoys. The decoys were generated by the DUD-E (Directory of Useful Decoys, Enhanced) web server.^75,76^ Both calculation analysis and figure generation were executed employing Maestro (v. 14.3).

### Molecular Dynamics

The structures of **1** complexed to sEH and 5-LOX from molecular docking were inputted for molecular dynamics simulations through a prior preparation using System Builder^77^ of Desmond: an orthorhombic box sized at 10 Å distance of the buffer from the solute, the OPLS4 force field, the TIP3P^78^ model of solvation, and Na^+^ and Cl^–^ ions for electroneutrality, adding supplementary NaCl (0.15 M). The prepared molecular systems were equilibrated through: 1) 100 ps of NVT (method = “Brownie”) MD run (10 K), restraining solute heavy atoms (50 kcal/mol);^79,80^ 2) 12 ps of NVT (method = Langevin) MD run (10 K), restraining solute heavy atoms (50 kcal/mol); 3) 0.12 ns of NPT (method = Langevin)^81^ MD run at 10 K, restraining solute heavy atoms (10 kcal/mol); 4) simulation of 0.12 ns of NPT (method = Langevin) at 310 K, restraining solute heavy atoms (10 kcal/mol); 5) no restrained atom simulation of 0.24 ns of NPT (method = Langevin) at 310 K. The equilibration phase of the two systems was evaluated using the Simulation Quality Analysis module of Desmond, examining pressure, volume, temperature, total, and potential energies. Unrestrained molecular dynamics (310 K, 100 ns) using the NPT (1.01 bar) ensemble class were executed, with 2.0 fs of integration time step and 1.2 ps of recording time.

### Cell-Free sEH Activity Assay

All compounds were initially screened for sEH inhibitory activity at single doses of 10 and 1 μM, tested in triplicate using the Soluble Epoxide Hydrolase Inhibitor Screening Assay Kit (10011671) purchased from Cayman and previously applied by us.^82,83^ For selected compounds, IC_50_ values were determined by testing serial 3-fold dilutions ranging from 60,000 nM to 0.45 nM. In brief, recombinant human sEH was diluted in 25 mM Bis-Tris buffer (pH 7) and preincubated for 15 min at 25 °C with the test compounds, the reference inhibitor AUDA, or the vehicle (2.5% DMSO). The enzymatic reaction was initiated by the addition of 5 μL of the substrate PHOME (10 μM in DMSO) to reach a final concentration of 0.25 μM. Upon hydrolysis of the epoxide moiety of PHOME by sEH, an intramolecular cyclization occurs, leading to the formation of a cyanohydrin intermediate. Under basic conditions, this intermediate decomposes into cyanide and 6-methoxy-2-naphthaldehyde, the latter being highly fluorescent. The fluorescence signal (λ_ex_ = 330 nm; λ_em_ = 465 nm) was measured using an EnSpire Multimode Plate Reader (PerkinElmer). The well-known sEH inhibitor, AUDA, was used as a positive control, obtaining an IC_50_ = 68.25 ± 0.27 × 10^–3^ μM.^83,84^

### 5-LOX UV-Based Assay

Colorimetric testing was conducted using 96-well flat-clear-bottom plates, with minor adjustments made to the FTC methodology as outlined by Lu et al.^85^ Using the proper buffer (Tris-HCl, 50 mM, pH 7.5), the recombinant lipoxygenase enzymes (ID: 60401) obtained from Cayman were diluted 10-fold. The protein was then preincubated with the test compounds, the reference compound (NDGA), or vehicle (2% DMSO) for 10 min. The enzymatic process was started by adding 5 μL of 2 mM linoleic acid solution, which was made from 100 mM linoleic acid in ethanol, activated with the same amount of NaOH (0.1 N), and diluted with H_2_O. This resulted in a final concentration of 100 μM. Using a Thermo Scientific Multiskan GO Microplate Spectrophotometer, Reagent I (4.5 mM FeSO_4_ in 0.2 M HCl) and Reagent II (3% NH_4_SCN methanolic solution) were employed as the FTC reagents to create a difference in the absorbance recorded at 480 nm. The well-known 5-LOX inhibitor, NDGA,^56^ was used as the positive control, obtaining an IC_50_ = 0.42 ± 0.02 μM (Figure S6).

### COX Fluorescence-Based Assay

The COX-1 inhibitor screening assay kit (Fluorometric-ab204698) and COX-2 inhibitor screening kit (Fluorometric-ab283401) were used to screen the generated compounds on the two proteins using COX-1 and COX-2. The procedure previously applied by us was followed to perform the assay.^86^ To achieve final concentrations of 10 and 1 μM (buffer with 2% DMSO) in the last well, the test or reference compounds (Indomethacin^87^ for COX-1 and Celecoxib^88^ for COX-2) were first dissolved in a stock solution of 100% DMSO and then diluted with buffer. The fluorescence at Ex/Em = 535/587 nm was found using an EnSpire Multimode Plate Reader (PerkinElmer, California, USA).

### Cell Assays

#### Reagents

Dimethyl sulfoxide (DMSO), 4-(2-hydroxyethyl)-1-piperazineethanesulfonic acid (HEPES), and lipopolysaccharide (LPS) from *Escherichia coli* O111:B4 were purchased from Sigma-Aldrich Co. (now under Merck, Darmstadt, Germany). Fetal bovine serum (FBS) and phosphate-buffered saline (PBS) were obtained from SIAL (Rome, Italy). All reagents not otherwise indicated were supplied by BioCell (Milan, Italy).

#### Cell Culture

Murine macrophage J774A.1 cells (AddexBio; Cat. No. C0003036) were cultured in 100 × 20 mm dishes (5 × 10^5^ cells/dish) in Dulbecco’s Modified Eagle’s Medium (DMEM; Corning; Cat. No. 10-013-CV) supplemented with 10% FBS (Sial; Cat. No. YourSIAL-FBS-SA), 100 U mL^–1^ penicillin, 100 μg mL^–1^ streptomycin (Corning; Cat. No. 30–002-CI), and 25 mM HEPES (Sigma-Aldrich; Cat. No. H0887). Cells were incubated at 37 °C in a humidified environment supplemented with 5% CO_2_.^89^

### Cytotoxicity of Monoterpene Phenol Dimers (1, 2, and 3) in Murine Macrophages

The cytotoxicity of compounds **1**–**3** on J774A.1 murine macrophage cells was assessed using the 3-(4,5-dimethyl-2-thiazolyl)­2,5-diphenyl-2H-tetrazolium bromide (MTT) assay following the experimental procedure reported by Saviano and colleagues.^42^ Briefly, J774A.1 cells (5 × 10^3^ per well) were seeded in 96-well plates and incubated overnight before exposure to compounds **1–3** (1, 3, 10, and 30 μM) for 24 h. Vehicle-treated cells (0.1% DMSO) were used as controls. MTT (BioBasic; Cat. No. T0793) solution (5 mg mL^–1^ in PBS; pH 7.4) was then added and, after 3 h of incubation, formazan crystals were dissolved in DMSO. Absorbance was recorded at 540 nm, and cell viability was determined by the following formula: OD of treated cells/OD of control × 100.

### Anti-Inflammatory Activity on Murine Macrophages

J774A.1 macrophages were cultured until reaching approximately 70% confluence. The culture medium was then replaced, and cells were co-treated for 24 h with LPS (10 μg mL^–1^, Sigma-Aldrich, Cat. No. L2630), compound **1** (3 and 10 μM), compound **2** (3 μM) (highest noncytotoxic concentrations identified via MTT assay), or the combination of both. After incubation, supernatants were harvested and stored at −80 °C for interleukin (IL)-6, prostaglandin E_2_ (PGE_2_), prostaglandin D_2_ (PGD_2_), 5-hydroxyeicosatetraenoic acid (5-HETE), leukotriene B_4_ (LTB_4_), 15-hydroxyeicosatetraenoic acid (15-HETE), and 12-hydroxyeicosatetraenoic acid (12-HETE) enzyme-linked immunosorbent assay (ELISA) analysis.^89^

### ELISA

The levels of IL-6 (R&D Systems; Cat. No. DY406), PGE_2_ (Abnova; Cat. No. KA0326), PGD_2_ (Elabscience; Cat. No. E-EL-006696T), 5-HETE (MyBioSource; Cat. No. MBS2602657), LTB_4_ (Elabscience; Cat. No. E-EL-0061), 15-HETE (MyBioSource; Cat. No. MBS755850), and 12-HETE (MyBioSource; Cat. No. MBS265806) in J774A.1 culture supernatants were quantified at 24 h using a commercially available ELISA kit, as previously described.^41^ Briefly, samples, standards, quality controls, and blank solutions were loaded onto antibody-coated microplates and processed according to the manufacturers’ instructions. Following the sequential incubation and washing steps, enzymatic color development was achieved using the appropriate substrate solution, and the reaction was terminated with stop solution. Absorbance was measured at 450 nm using a microplate reader (Multiskan GO Microplate Spectrophotometer; Thermo Scientific). Antigen concentrations in the samples were calculated from the corresponding standard curve, normalized to the sample volume, and expressed as pg mL^–1^.

## Supplementary Material



## Notes

Superimposition of **1**–**3** docked poses with the X-ray conformation of AKBA, ^1^H NMR spectra for compounds **1**–**3**, IC_50_ curves for compounds **1** (A) and **2** (B) on sEH and 5-LOX, and percentage of COX inhibition (PDF)
