# Supplementary material for: Anti-Inflammatory Activity of Monoterpene Phenol Dimers from Satureja bachtiarica
Source: ACS Omega. 2026 Sep 7;11(37):55272–85. doi: 10.1021/acsomega.6c03194 (PMC13613392; doi:10.1021/acsomega.6c03194)
Supplement: Supplementary file 1 [file ao6c03194_si_001.pdf]

Supporting Information

Anti-inflammatory activity of monoterpene phenol dimers from *Satureja bachtiarica*

Simone Di Micco, Milena Masullo, Noemi Marigliano,* Ester Colarusso, Marzieh Rahmani Samani, Anna Schettino, Anella Saviano, Francesco Maione, Sonia Piacente,* and Giuseppe Bifulco*

Contents

Figure S1. Superimposition of **1-3** docked poses with X ray conformation of AKBA.

Figure S2. ^{1}H NMR Spectrum (600 MHz, CD_3OD) of compound **1**.

Figure S3. ^{1}H NMR Spectrum (600 MHz, CD_3OD) of compound **2**.

Figure S4. ^{1}H NMR Spectrum (600 MHz, CD_3OD) of compound **3**.

Figure S5. IC_{50} curves for compounds **1** (A) and **2** (B) on sEH.

Figure S6. IC_{50} curves for compounds **1** (A) and **2** (B) and NDGA (C) on 5-LOX.

Table S1. Percentage of cyclooxygenases (COX-1 and COX-2) inhibition.

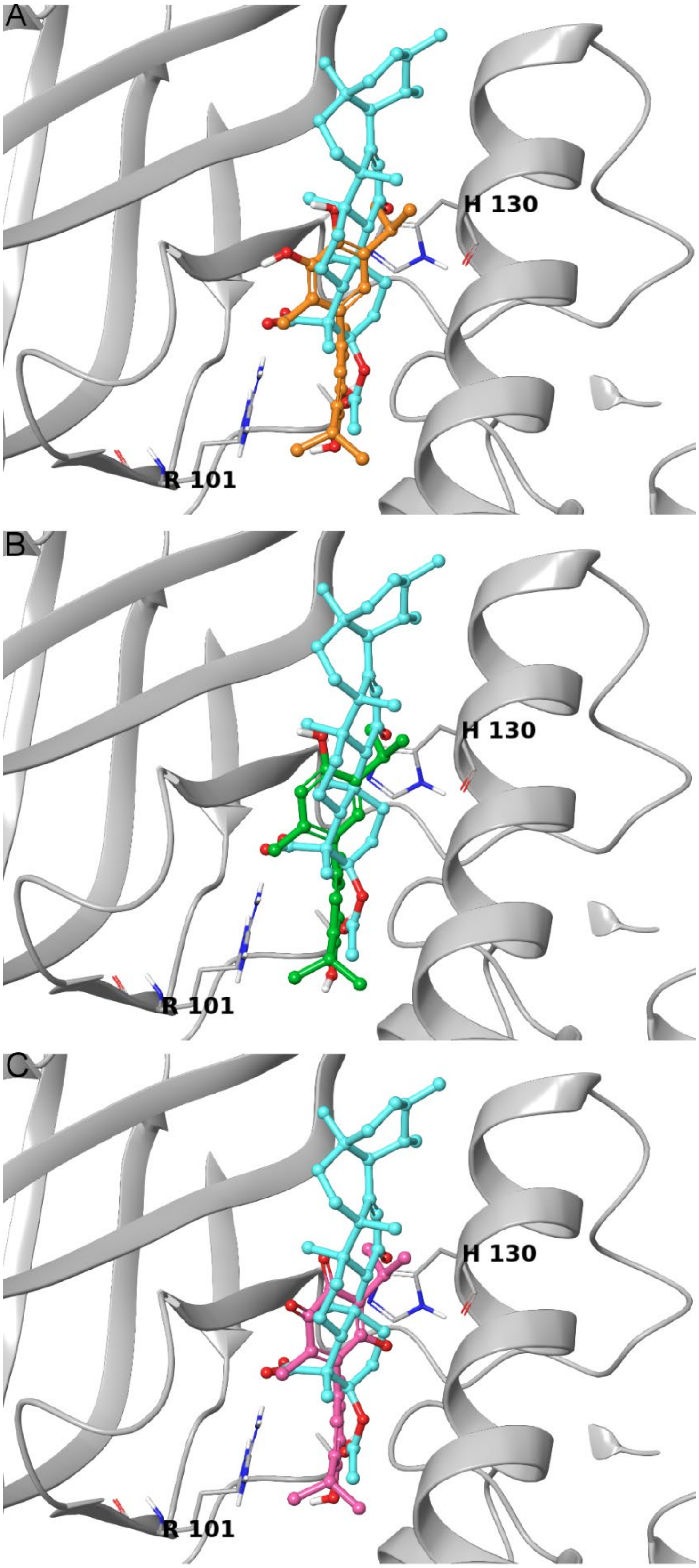

Figure S1. Superimposition of **1**-**3** docked poses, respectively (A-C) with Xray conformation of AKBA (PDB ID: 6CFN). 5-LOX is by grey (B) tube with the atom color code: C, as for tube; polar H, white; N, dark blue; O, red; S, yellow). The ligands are represented by sticks and balls (with the atom color code used for the protein except for C: orange for **1**, green for **2**; faded salmon for **3**, cyan for AKBA).

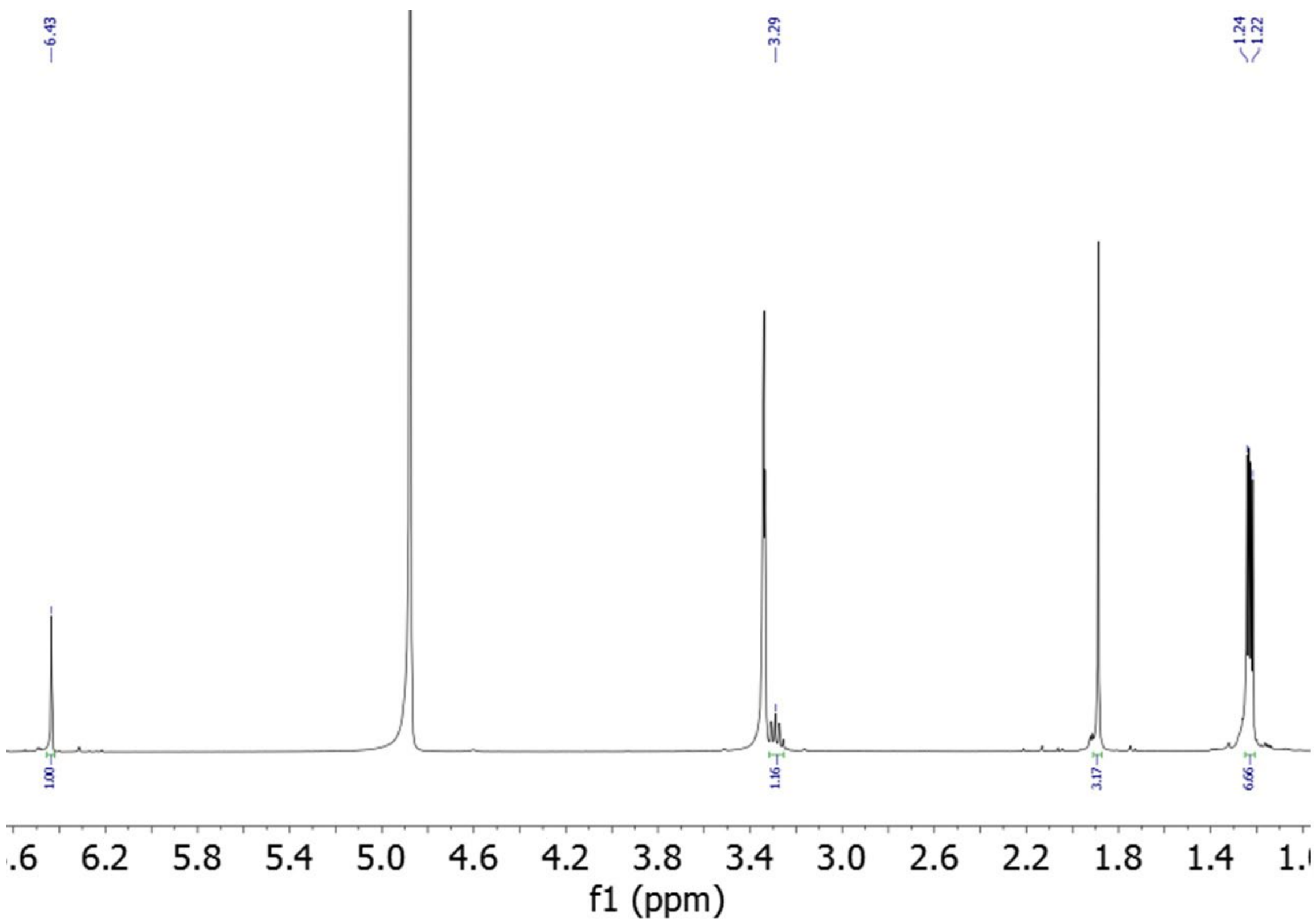

Figure S2. ^{1}H NMR Spectrum (600 MHz, CD_3OD) of compound **1**.

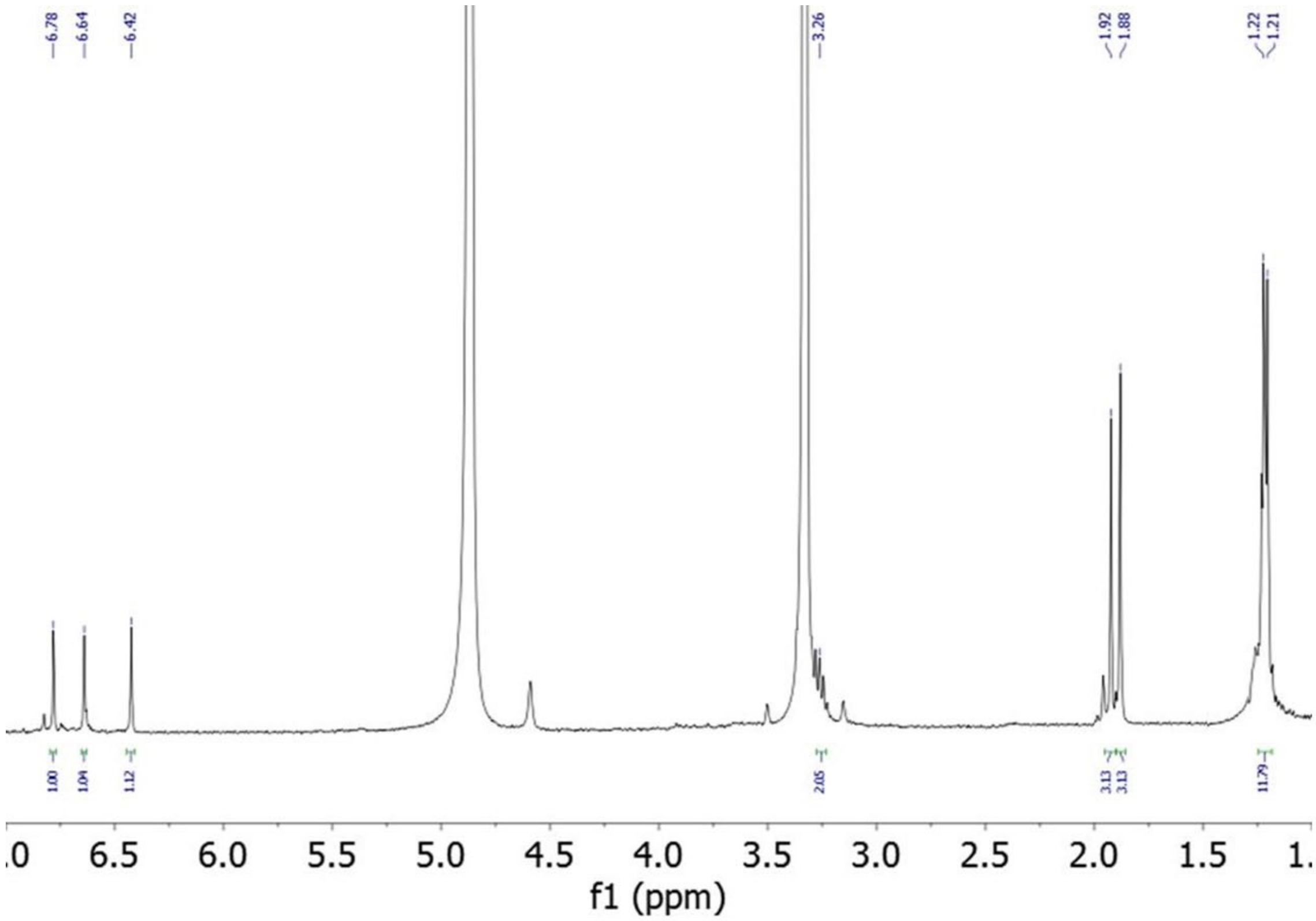

Figure S3. ^{1}H NMR Spectrum (600 MHz, CD_3OD) of compound **2**.

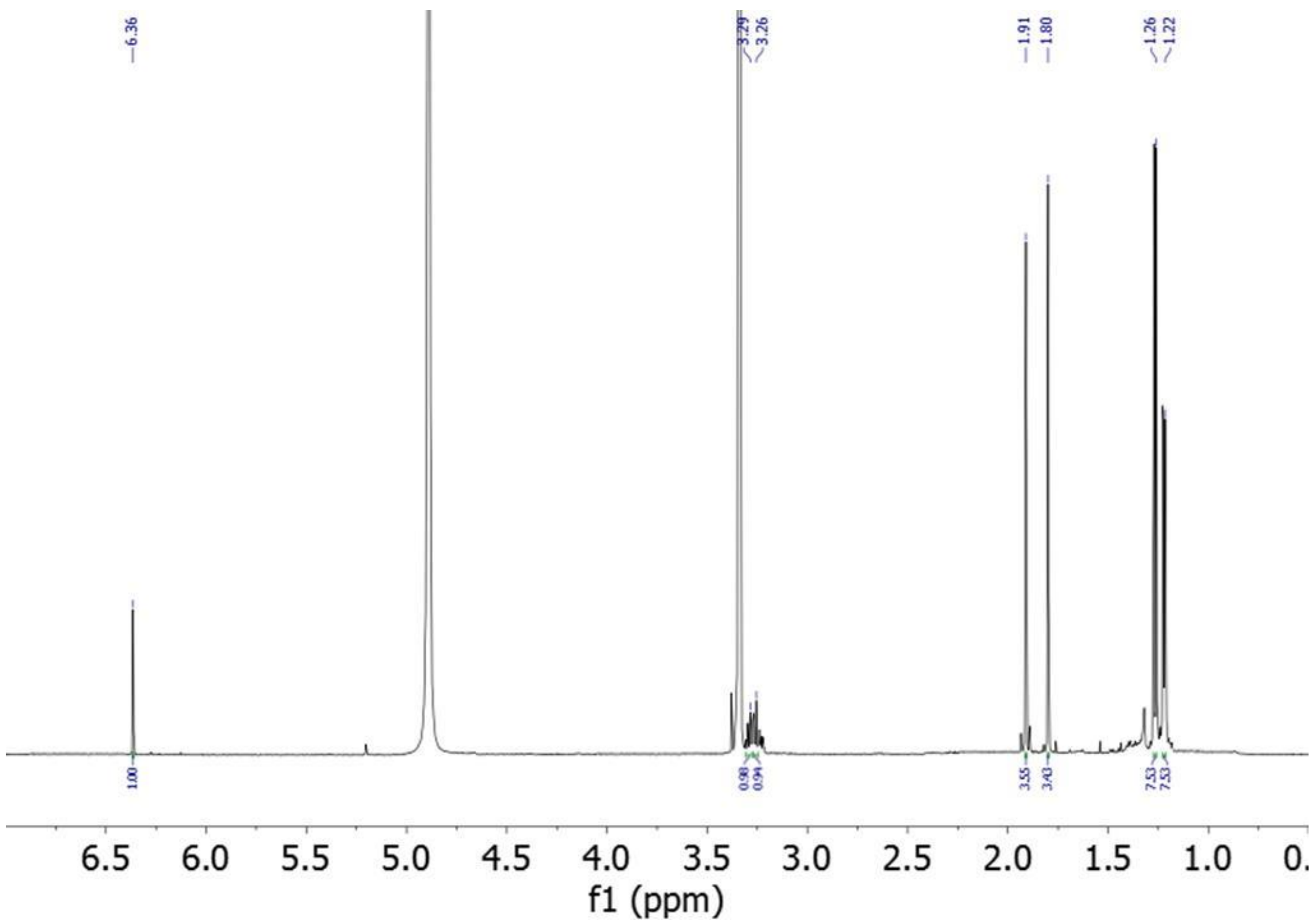

Figure S4. ^{1}H NMR Spectrum (600 MHz, CD_3OD) of compound **3**.

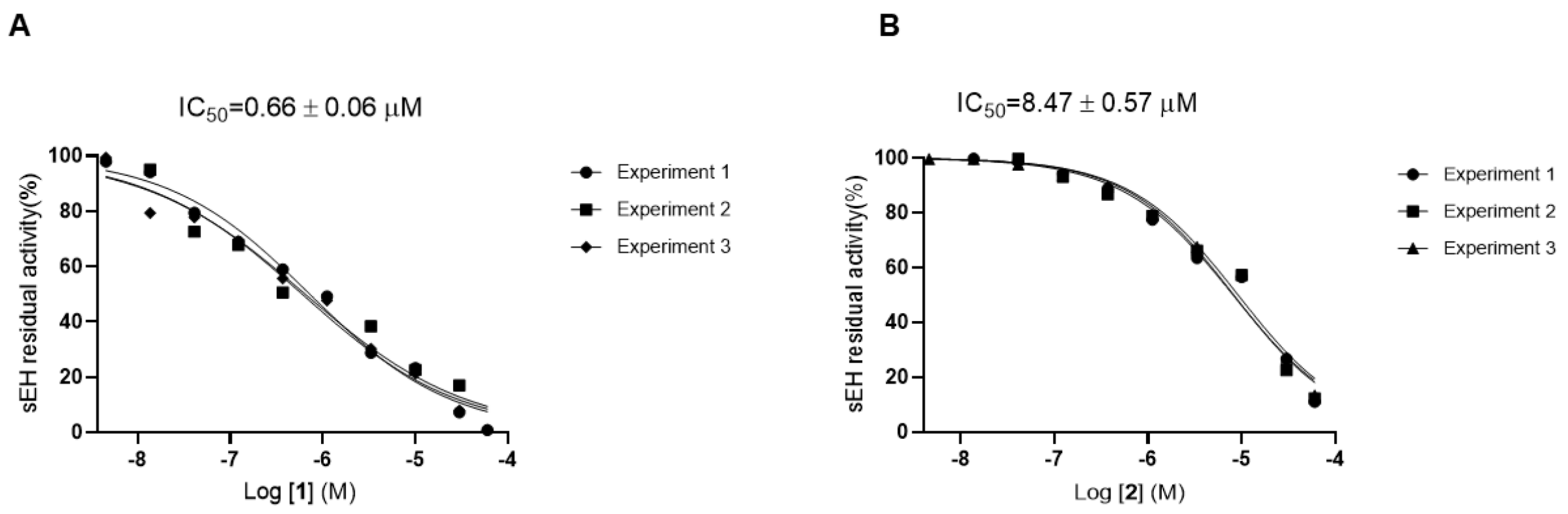

Figure S5. IC_{50} curves for compounds **1** (A) and **2** (B) on sEH. Data are expressed as means of the three experiments ± SD.

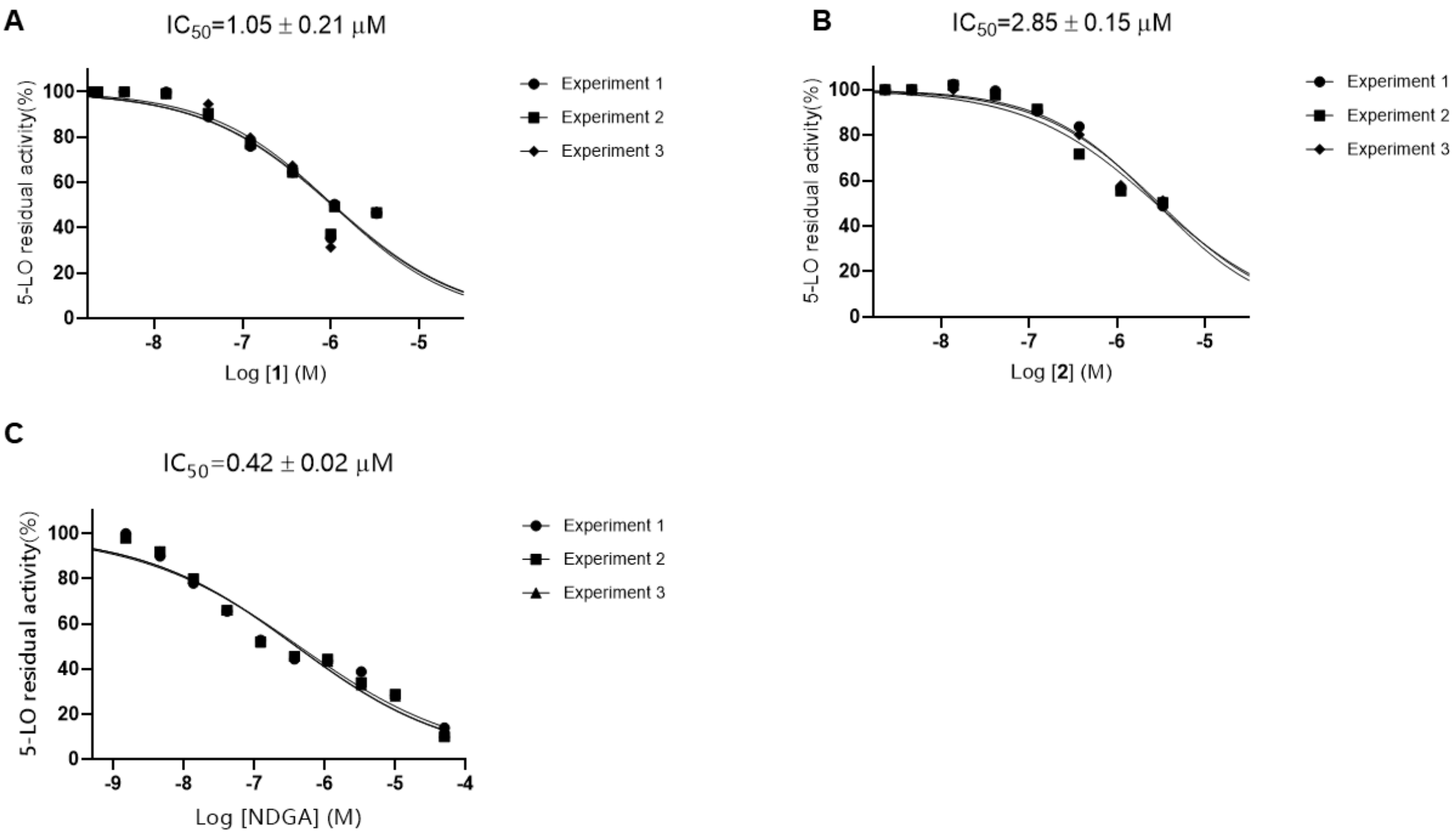

Figure S6. IC_{50} curves for compounds **1** (A), **2** (B) and NDGA(C) on 5-LOX. Data are expressed as means of the three experiments ± SD.

Table S1. Percentage of Cyclooxygenases (COX-1 and COX-2) inhibition after incubation with compounds **1-3** at 10 and 1 µM and related IC_{50} values. Data are expressed as a percentage of control (100%), n=3. Indomethacin (COX-1 known inhibitor) and Celecoxib (COX-2 known inhibitor) were used as reference compounds.

	COX-1		COX-2	
compound	% inhibition at 10 µM of compound	% inhibition at 1 µM of compound	% of inhibition at 10 µM of compound	% inhibition at 1 µM of compound
**1**	3.2 ± 2.5	7.9 ± 5.6	~0	10.7 ± 1.5
**2**	3.1 ± 1.4	16.7 ± 5.0	~0	8.3 ± 2.4
**3**	4.2 ± 0.2	23.6 ± 3.4	~0	7.4 ± 0.9
Indomethacin	81.1 ± 0.2	\	\	\
Celecoxib	\	\	85.2 ± 2.3	\
